# Microfluidic model of ductal carcinoma in situ with 3D, organotypic structure

**DOI:** 10.1186/s12885-015-1007-5

**Published:** 2015-01-21

**Authors:** Lauren L Bischel, David J Beebe, Kyung E Sung

**Affiliations:** 1 Department of Biomedical Engineering, Wisconsin Institutes for Medical Research, University of Wisconsin, Madison, WI USA; 2 The University of Wisconsin Carbone Cancer Center, University of Wisconsin, Madison, WI USA

**Keywords:** Mammary duct, Breast cancer, Ductal carcinoma in situ, Microfluidics, 3D, Lumen, Microenvironment, Extracellular matrix

## Abstract

**Background:**

Ductal carcinoma in situ (DCIS) is a non-invasive form of breast cancer that is thought to be a precursor to most invasive and metastatic breast cancers. Understanding the mechanisms regulating the invasive transition of DCIS is critical in order to better understand how some types of DCIS become invasive. While significant insights have been gained using traditional in vivo and in vitro models, existing models do not adequately recapitulate key structure and functions of human DCIS well. In addition, existing models are time-consuming and costly, limiting their use in routine screens. Here, we present a microscale DCIS model that recapitulates key structures and functions of human DCIS, while enhancing the throughput capability of the system to simultaneously screen numerous molecules and drugs.

**Methods:**

Our microscale DCIS model is prepared in two steps. First, viscous finger patterning is used to generate mammary epithelial cell-lined lumens through extracellular matrix hydrogels. Next, DCIS cells are added to fill the mammary ducts to create a DCIS-like structure. For coculture experiments, human mammary fibroblasts (HMF) are added to the two side channels connected to the center channel containing DCIS. To validate the invasive transition of the DCIS model, the invasion of cancer cells and the loss of cell-cell junctions are then examined. A student *t*-test is conducted for statistical analysis.

**Results:**

We demonstrate that our DCIS model faithfully recapitulates key structures and functions of human mammary DCIS and can be employed to study the mechanisms involved in the invasive progression of DCIS. First, the formation of cell-cell junctions and cell polarity in the normal mammary duct, and the structure of the DCIS model are characterized. Second, coculture with HMF is shown to induce the invasion of DCIS. Third, multiple endpoint analyses are demonstrated to validate the invasion.

**Conclusions:**

We have developed and characterized a novel in vitro model of normal and DCIS-inflicted mammary ducts with 3D lumen structures. These models will enable researchers to investigate the role of microenvironmental factors on the invasion of DCIS in more in vivo-like conditions.

## Background

Ductal carcinoma in situ (DCIS) is a non-invasive form of breast cancer that originates and is confined to the lumen of the mammary duct [1,2]. Even though DCIS itself is non-invasive, there has been a dramatic increase in the reporting of DCIS due to the enhancement in imaging technologies and increased rate of breast cancer screenings. It is known that some cases of DCIS will never become invasive; however, roughly 30% of DCIS cases will progress to IDC within 10 years of diagnosis if left untreated [3]. Recent studies have shown that the DCIS and IDC share similar risk factors and present similar gene expression patterns. This makes it nearly impossible to distinguish which DCIS cases are more aggressive and will progress to IDC based on genetics alone [4]. This inability to distinguish indolent from aggressive DCIS results in a clinical dilemma balancing the potential benefits of reducing the risk of a future invasive breast cancer versus the risks of unnecessary treatments such as surgery, radiation therapy, and endocrine therapy [5,6].

Due to the risks associated with overdiagnosis and overtreatment of DCIS patients, there is increased interest in studying which factors promote the invasive transition of DCIS to IDC. To this end, several in vitro and in vivo models of DCIS have been developed. The MINO (mammary intraepithelial outgrowth) mouse model and the MIND (mammary intraductal) mouse model are promising in vivo models of DCIS. In the MINO model, mice have been engineered to have neoplastic outgrowths from mammary ducts [7,8] while in the MIND model, human DCIS cell lines are transplanted intraductally to mimic the structure of DCIS found in humans [9,10]. The in vivo models such as these are more physiologically relevant and allow us to investigate the influence of various microenvironmental factors because the models use a whole organism. However, in vivo models can be time-consuming and expensive, limiting their use in routine screens investigating potential factors capable of promoting the invasive transition of DCIS. In addition, the use of mouse models often presents ethical issues and, more importantly, the mouse mammary microenvironment does not faithfully recapitulate the human mammary microenvironment [11]. For instance, human breast tissue has more fibrous connective tissue and less adipocytes than mouse mammary tissue.

Various in vitro culture models have been introduced to investigate the effect of microenvironmental factors on the fate of DCIS cells. First, model cell lines have been developed to look at the influence of molecular changes in the breast cancer tumor microenvironment in vitro [12,13]. Second, traditional 3D macroscale models in multi-wells or transwells incorporating 3D extracellular matrix (ECM) and coculture with other cell types have been developed and used to investigate the effects of the ECM and interactions between tumor cells and stromal cells on invasion [13,14]. Third, with the advancement in microscale technology, microscale 3D DCIS models have also been recently developed. Microscale models enhance functionality and efficiency while maintaining key attributes of their macroscale counterparts. By taking advantage of microfabrication technology, the geometry of the microsystem is varied to enhance temporal and spatial controls [15,16]. In fact, a recent microscale 3D model developed to investigate the invasive progression of DCIS revealed the importance of the distance between cancer cells and fibroblasts as well as the presence of heterogeneous contact between cancer cells and fibroblasts in regulating the invasive progression of DCIS [15]. While these models have made important advances, there is a lack of models with physiologically relevant lumen structures which have been shown to influence cell behavior [17-19]. Additionally, recent evidence supports the ability of in vitro tissue structure to influence the behavior of other cell types and the outcome of biological studies [19]. For instance, using micro-tissue patterns, Nelson et al. have found that tissue geometry determines the sites of mammary branching morphogenesis due to the distribution of secreted factors and mechanical stresses [20]. In addition, it was shown that culturing epithelial cells in different 2D patterns and initialing EMT influences the spatial pattern of EMT [21]. Based on the influences of tissue geometry observed in these examples, it is important to develop in vitro models with improved physiologically relevant tissue structure in order to complement and overcome the limitations inherent in current in vivo and in vitro models and improve researchers’ ability to effectively study mechanisms governing the invasive transition of DCIS.

Here, we present a novel 3D in vitro mammary duct model with physiologically relevant lumen structures. Taking our cue from the MIND mouse model described above, we have developed a DCIS model by filling the mammary duct models with DCIS cells in order to study the effects of microenvironmental factors on the invasive transition of DCIS. Using a viscous finger patterning method [22,23], lumen structures are patterned with a double-layered ECM hydrogel consisting of a collagen I outer layer with a Matrigel inner layer to model the basement membrane. The lumens are lined with a non-cancerous human mammary epithelial cell line, MCF10a, to model a normal mammary duct. To model a mammary duct with DCIS, MCF10aDCIS.com cells (MCF10a-derived DCIS cell line) are pipetted into the lumen of the in vitro mammary duct where they grow and fill the lumen. Because our system provides a more in vivo-like structure of DCIS (i.e., a mammary epithelial cell-lined lumen filled with cancer cells), the DCIS cells present a more in vivo-like invasive transition when the cancer cells are cocultured with stromal fibroblasts. In traditional methods, cancer cells are cultured from a single cell and form elongated clusters when they become invasive. However, in our system, the mammary duct already contains numerous cancer cells which then invade from the duct out into the stroma, much like they would in vivo. The viscous fingering method enables users to easily pattern lumen structures through ECM hydrogels in microchannels using only a micropipette, is highly compatible with high-throughput molecular screening experiments using automated pipeting systems.

## Methods

### Device fabrication

**Figure 1:**
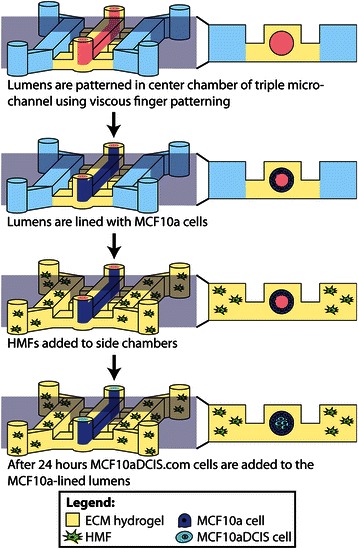
**Schematic overview of model patterning methods.** As previously described, a lumen can be patterned through a collagen I hydrogel in the center chamber of a triple microchannel using viscous finger patterning [22]. The collagen I lumen is then coated with a Matrigel lining. Following polymerization, MCF10a cells are used to line the lumen and mimic a mammary duct structure. HMF cells in a collagen I hydrogel can be added to the side chambers. To model DCIS, MCF10aDCIS.com cells are flowed into the lumen after 24 hours.

Polydimethylsiloxane (PDMS, Sylgard 184 Silicone Elastomer Kit, Dow Corning, Midland, MI) elastomer base and curing agent were mixed at a 10:1 ratio and degassed for 45 minutes under vacuum at room temperature. The degassed PDMS was then poured over SU-8 master molds that were generated using standard soft lithography methods [24]. PDMS was cured at 80 degrees Celsius for 4 hours. Single and triple (Figure 1) microchannel geometries that have been previously described [23] were used in these experiments depending on the application. Briefly, single microchannels have a width of 500 μm, height of 500 μm, and length of 5 mm with inlet and outlet ports on each end. Triple microchannels consist of three single microchannels connected by smaller diffusion channels that are 100 μm in height, 400 μm in width and 500 μm in length.

### Cell culture

Human mammary epithelial cells (MCF10a) were obtained from ATCC (Manassas, VA, USA) and maintained with DMEM/F12 medium (Invitrogen, Carlsbad, CA, USA) supplemented with 5% horse serum (Invitrogen, Carlsbad, CA, USA), 20 ng/ml epidermal growth factor (EGF, Peprotech, Oak Park, CA, USA), 0.5 mg/ml hydrocortisone (Sigma-Aldrich, St. Louis, MO, USA), 100 ng/ml cholera toxin (Sigma-Aldrich, St. Louis, MO, USA), 10 μg/ml insulin (Sigma-Aldrich, St. Louis, MO, USA) and 1% Pen/Strep (Invitrogen, Carlsbad, CA, USA) on regular tissue culture flasks. MCF10a-DCIS.com (MCF10aDCIS) cells were obtained from Asterand (Detroit, MI, USA) and maintained in DMEM/F12 medium (Invitrogen, Carlsbad, CA, USA) supplemented with 5% horse serum (Invitrogen, Carlsbad, CA, USA) and 1% Pen/Strep (Invitrogen, Carlsbad, CA, USA) on regular tissue culture flasks. Human mammary fibroblasts (HMFs) were obtained from Dr. Charlotte Kuperwasser (originally located at Whitehead Institute for Biomedical Research, Nine Cambridge Center, Cambridge, MA) and maintained in DMEM supplemented with 10% fetal bovine serum (FBS, Invitrogen, Carlsbad, CA, USA) and 1% Pen/Strep (Invitrogen, Carlsbad, CA, USA) on regular tissue culture flasks [25].

### Hydrogel preparation

For lumen patterning as well as for culturing fibroblasts, a hydrogel consisting of ECM proteins made of a final concentration of 6.0 mg/ml Type I collagen (rat tail, BD Biosciences, Bedford, MA, USA. To make roughly 125 μl of hydrogel solution, 1.56 μl of a basic solution (5.0 N NaOH), 20 μl of culture medium and 25 μl of 5X PBS was added to 78 μl of the collagen I and incubated on ice for a period between 5–10 minutes to apply an additional nucleation phase [26]. These volumes were scaled up to prepare the amount of hydrogel solution needed for a given experiment. Matrigel (BD Biosciences, Bedford, MA, USA) was also used as a coating material (described in detail below).

### Lumen patterning

Prior to patterning lumens, devices were oxygen-plasma-treated to bond the PDMS channels to a glass surface (the inside of a glass-bottom Petri dish, MatTek, Ashland, ME, USA), and to render the inside of the chambers hydrophilic. The devices were coated with 100 μg/ml FN to facilitate adhesion of the hydrogel to the channel walls. After 20 min incubation at room temperature, the FN solution was aspirated from the channel. To pattern a lumen through a hydrogel in a single microchannel, viscous finger patterning was used as previously described [22]. Briefly, viscous finger patterning is performed by dispensing 5 μl of a pre-polymerized hydrogel solution into a microchannel. Then a small droplet of a low viscosity fluid (roughly 2 μl of culture medium) is dispensed through the hydrogel solution via surface tension-based passive pumping (i.e. fluid flows from the inlet port to the outlet port due to differences in surface tension) [27]. Due to differences in viscosity, when the less viscous medium flows through the channel, it pushes through the more viscous hydrogel solution in a finger-like pattern leaving behind a medium-filled lumen in the hydrogel. After incubating the hydrogel in the microchannels for 10 min at 37°C, polymerization of the hydrogel is completed, resulting in a patterned lumen through a hydrogel. In these experiments an outer layer of 6.0 mg/ml Collagen I was patterned. After 10 min incubation at 37°C, Matrigel was added to the lumen and viscous fingering was performed again to pattern a lumen through this inner layer.

For coculture experiments, a single lumen in the center channel of a triple microchannel was patterned, leaving the two side channels open. Lumen patterning was achieved by the method described in Figure 1.

### Mammary epithelial cell seeding

To line the lumen with MCF10as and generate a normal mammary duct model, a cell solution was prepared at 50,000-60,000 cells/μl and 2 μl were added to the lumens. With the lid of the Petri dish securely attached, the dish containing the microchannels was taped to the rod that is attached to a motor (BBQ Rotisserie Variable Speed Reversible Brushless Gear Motor, Wondermotor, CA, USA). This motor was placed inside an incubator at 37 degrees Celsius and tuned to rotate the dishes at 2 RPM for 30 minutes to allow cell attachment. After this procedure, channels were righted and incubated at 37 degrees Celsius for a minimum of 2 hours before being perfused with fresh medium via passive pumping to rinse unattached cells.

### DCIS cell seeding

To generate the DCIS model, lumens were first lined with MCF10a cells and cultured overnight at 37 degrees Celsius. Then, 2 μl of a cell solution with 50,000 MCF10aDCIS cells/μl was added to the lumens. With the lid of the Petri dish securely attached, the dish containing the microchannels was taped to the rod that is attached to a motor (BBQ Rotisserie Variable Speed Reversible Brushless Gear Motor, Wondermotor, CA, USA). This motor was placed inside an incubator at 37°C and tuned to rotate the dishes at 2 RPM for 30 minutes to enable cells to attach evenly throughout the lumen. Alternatively, it is possible to achieve an even layer of cells by manually rotating the dish by 90 degrees every 15 minutes, reseeding cells into the channel between each rotation [23]. After this procedure, channels were incubated at 37 degrees Celsius for roughly 2 hours, rinsed with fresh medium to remove unattached cells and incubated at 37 degrees Celsius over night. In control experiment, MCF10a cells replaced the DCIS cells to fill the lumens.

### HMF cell seeding for DCIS invasion experiments

To assess the ability of fibroblasts to induce the invasive transition of DCIS to IDC, the DCIS lumen models were patterned in the center channel of a tri-channel device and cocultured with fibroblasts in both of the side channels. 24 hours after the addition of MCF10as lining the lumens, a solution with a final concentration of 6.0 mg/ml collagen I and 6,000 HMF cells/μl was added to the side channels. The MCF10aDCIS cells were then added as previously described. Control channels with MCF10a cells alone, MCF10a cells with HMFs and the DCIS lumen model (MCF10a lining with MCF10aDCIS cells) alone were also patterned. Bright-field images were collected daily for a period of 5 days to assess invasion of the mammary duct and DCIS lumen models.

### Cell staining

To confirm that MCF10a cells formed monolayers coating the entire lumen, and that the cells were forming cadherin junctions, mammary ducts were cultured for 48 h, fixed with 4% paraformaldehyde (PFA) (Fisher Scientific, Hampton, NH, USA), and stained for e-cadherin. Models were blocked with 3% BSA, immunolabeled with mouse anti-human e-cadherin antibodies (5 μg/ml, BD Biosciences, Bedford, MA, USA), and fluorescently labeled with AlexaFlour 488 (10 μg/ml, anti-mouse, Molecular Probes, Eugene, OR, USA). Hoechst 33342 (10 μg/ml, Molecular Probes) was used to stain the nuclei. To confirm that the MCF10a cells were properly polarizing in the mammary ducts, cells were cultured for 72 hours, fixed and blocked as previously described. The cells were then stained for basement membrane protein laminin-5 (10 μg/ml, mouse, human specific, AbCam, Cambridge, MA, USA), apical marker GM130 (10 μg/ml, rabbit, AbCam, Cambridge, MA, USA) and nuclei (Hoechst 33342). The laminin was fluorescently labeled with AlexaFlour 488 (10 μg/ml, anti-mouse, Molecular Probes, Eugene, OR, USA) and the GM130 was fluorescently labeled with AlexaFlour 647 (10 μg/ml, anti-rabbit, Molecular Probes, Eugene, OR, USA). To visualize the DCIS lumen model, MCF10a cells were stained with green cell tracker dye (10 μM, Life Technologies, Grand Island, NY, USA) and MCF10aDCIS cells were stained with blue cell tracker dye (10 μM, Life Technologies, Grand Island, NY, USA) for 30 minutes prior to each respective cell seeding. The channels were then fixed after 72 hours as previously described. For multiphoton laser scanning microscopy and second harmonic generation imaging, cells were visualized by staining for filamentous actin using Alexa Flour 594 phalloidin (10 μg/ml, Invitrogen).

### Image acquisition

Fluorescently labeled samples were imaged using an A1RSi confocal microscope (Nikon Instruments, Tokyo, Japan) and images were acquired using NIS Elements Advanced software (Nikon). Images files were converted using Fiji software, and volume-rendered images were generated using OsiriX. Bright-field images were collected with an Olympus IX70 microscope (Center Valley, PA, USA) and acquired using MetaMorph 7.5 (Molecular Devices, LLC, Sunnyvale, CA, USA). Multiphoton laser scanning microscopy (MPLSM) imaging was performed on an Optical Workstation that was constructed around a Nikon Eclipse TE300. A MaiTai Deepsee Ti: sapphire laser (Spectra Physics, Mountain View, CA) excitation source tuned to 890 nm was used. The beam was focused onto the sample with a Nikon (Mehlville, NY) 10X SuperFluor air-immersion lens (numerical aperture (NA) = 0.5). All SHG imaging was detected from the back-scattered SHG signal with a H7422 GaAsP photomultiplier detector (Hamamatsu, Bridgewater, NJ), and the presence of collagen was confirmed by filtering the emission signal with a 445 nm (narrow-band pass) filter (TFI Technologies, Greenfield, MA) to isolate the SHG signal. Images were acquired using WiscScan (http://www.loci.wisc.edu/software/wiscscan), a laser scanning software acquisition package developed at LOCI (Laboratory for Optical and Computational Instrumentation, University of Wisconsin, Madison, WI) described in previous work [15].

### Invasion experiment analysis

To investigate invasiveness bright field images of three independent experiments were analyzed manually using ImageJ for invasion frequency and area of invasive lesions. The entire length of the microchannel was analyzed and the ports were excluded. A Student’s *t*-test was used to determine significant differences between samples. P-values less than 0.05 were considered to be significant. To analyze collagen modification from the SHG images, the average intensity of a 20 pixel region extending from the cell-ECM boundary was measured using ImageJ. The intensity of regions surrounding the non-invasive cells were compared to the region surrounding the invasive cells.

## Results and discussion

### Lumen-based in vitro mammary duct model

A microfluidic model of a normal mammary duct with a physiologically relevant lumen structure was developed as a foundation for a lumen-based DCIS model. To generate the model, viscous finger patterning [22,23] was used to pattern a lumen through a double-layered hydrogel consisting of an outer layer of collagen I and an inner layer of Matrigel to represent the basement membrane. Briefly, microchannels with two ports open to the air are filled with a pre-polymerized solution of collagen I. Surface tension-driven pumping (i.e. passive pumping) is used to flow a droplet of a culture medium from the inlet port to the outlet port [27]. Due to the differences in viscosity between the hydrogel and the medium, the medium flows through the center of the channel patterning a lumen through the hydrogel [22]. Following polymerization of the collagen gel, Matrigel is flowed into the lumen and rinsed with culture media to model the basement membrane and provide a thin coating and provide proteins, such as laminin and collagen IV, required for normal epithelial cell polarization [28]. After polymerization of the Matrigel, lumens were then lined with MCF10a cells, a non-cancerous human mammary epithelial cell line (Figure 1). We found that the presence of the basement membrane in our system enhanced the attachment of MCF10a cells as well as the formation of a confluent monolayer in the lumen and polarization of the cells. While there are other methods to pattern lumen structures through ECM hydrogels for modeling mammary ducts [20] or other structures with lumen geometries (i.e. vessels) [29,30], the use of viscous finger patterning offers several advantages. This technique is easy to perform and requires only the use of a micropipette, making this technique accessible for widespread use. Additionally, this technique can be performed with automated liquid handling systems and scaled up into high-throughput screening arrays [31,32].

**Figure 2:**
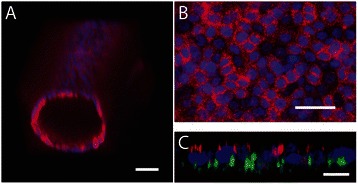
**Mammary duct model with 3D lumen structure. A)** Volume-rendered confocal image of MCF10a cells lining a lumen patterned through a collagen I hydrogel and coated with Matrigel. Cells were cultured for 72 hours and then stained for e-cadherin (red) and nuclei (blue). Scale bar represents 50 μm. **B)** Confocal slice of bottom of the lumen to show e-cadherin localization around cell junctions. Scale bar represents 5 μm. **C)** Volume-rendered confocal image of MCF10a cells lining a lumen and stained for laminin V (green), nuclei (blue) and GM130 (red) to demonstrate cell polarization. Scale bar represents 1 μm.

The formation of adherens junctions between neighboring cells and proper apico-basal cell polarity are essential for normal epithelial organization [33-35]. To confirm that our mammary duct model was demonstrating proper junction formation cells were stained for a known epithelial cell junction marker, e-cadherin (Figure 2A-B). The MCF10A cells in our channel showed adherens junctions throughout the lumen structure. To confirm that the MCF10a cells were exhibiting proper apical-basal polarity, the cells were cultured for 72 hours and stained for an apical polarity marker, GM130, and a basal polarity marker, laminin-5. As shown in the Figure 2C, the apical golgi markers were facing inward toward the inside of the hollow lumen and the basal marker were facing outward. A human specific antibody was used for laminin-5 to ensure that the laminin was produced by the MCF10a cells and not part of the mouse-derived Matrigel. This was confirmed by staining an unlined lumen with the same antibody; no cross-reactivity was detected. It has been reported that MCF10a cell lines do not usually form tight junctions, reaching an incomplete differentiation stage [36]. Thus, we did not stain for the formation of tight junctions in our model.

### Modeling Ductal Carcinoma in situ

**Figure 3:**
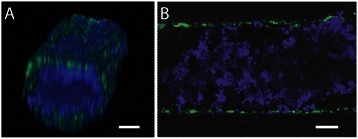
**To model ductal carcinoma in situ MCF10aDCIS.com cells were added to the center of MCF10alined lumens after 24 hours.** Volume-rendered **(A)** and confocal slice **(B)** images are shown. Cells were stained with cell tracker green (MCF10a) and cell tracker blue (MCF10DCIS.com) and cultured for 72 hours after the addition of the DCIS cells. Scale bars represent 100 μm.

To better mimic the DCIS structure found in vivo we used the model of the normal mammary duct as the foundation. Following MCF10a cell seeding, epithelium was cultured for 24 hours before pipetting MCF10aDCIS cells into the lumens (Figure 1). Figure 3 shows confocal images of the DCIS model 48 hours after MCF10aDCIS cell seeding where the non-neoplastic MCF10a cells were labeled with cell tracker green and the MCF10aDCIS cells were labeled with cell tracker blue. Complementary to recent in vitro mammary duct models which focus on studying the initiation of mammary tumors within the mammary duct from small groups of cancer cells [35,37], we have used a high number of DCIS cells in order to mimic a more advanced stage of DCIS, similar to the MIND mouse model that has been previously developed [10]. The MCF10aDCIS cell line was derived as a model of comedo-type DCIS [12], and it was observed that the MCF10a cells completely filled the center of the lumen. This is similar in structure to comedo-type DCIS found in humans.

**Figure 4:**
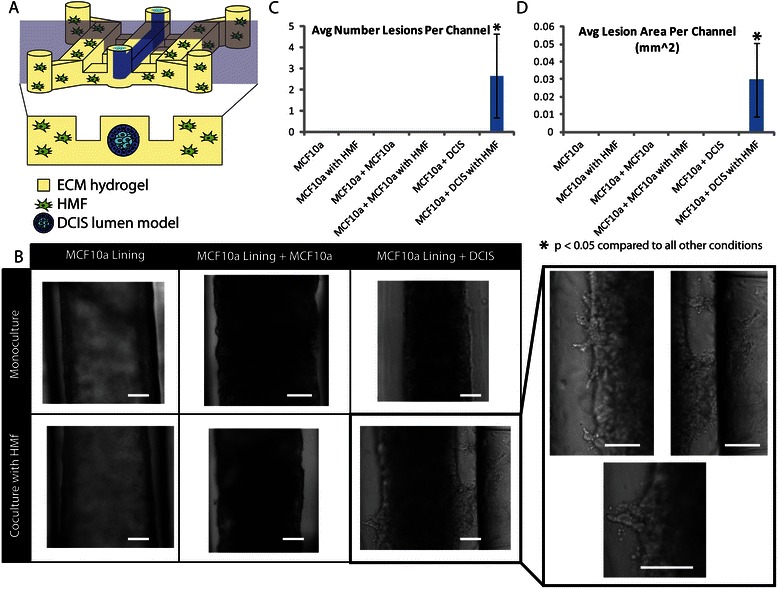
**Coculture with HMF induces invasive transition in the DCIS model. A)** Schematic image of coculture experiments with the DCIS model in the center chamber of a triple microchannel and HMFs in the side chambers. **B)** Bright-field images of mammary duct, mammary duct filled with MCF10a cells, and mammary duct filled with DCIS cells either alone or in coculture with HMFs. Scale bars represent 100 μm (main chart) and 50 μm (close up image at the right). The average number **(C)** and area **(D)** of invasive lesions in the different culture conditions were quantified across three independent experiments with at least three channels per experiment. A student Student’s *t*-test was used to determine significant differences between culture conditions (p < 0.05 is considered to be significant).

Evidence suggests that the invasive progression of DCIS to IDC is strongly influenced by alterations in the surrounding stromal cells and extracellular matrix [16,38,39]. To show that our model can be used to study such paracrine interactions, we have cocultured our DCIS model with human mammary fibroblasts (HMF), stromal cells that have been shown to induce the invasive transition in our previous work by Sung et al. [15]. This cell line was isolated from fibrocystic tissue and is not tumorigenic [25]. However, protein expression profiling shows that the expression pattern of these cells overlaps to some degree with the pattern of cancer-associated mammary fibroblasts [40]. For the coculture experiments, we used a system containing three larger microchannels connected by smaller diffusion channels [23]. The physical separation by diffusion channels simplified the loading process and also provided more in vivo-like cell organization by putting stromal fibroblasts next to mammary ducts. We have previously shown that diffusion channels between two cell compartments enabled paracrine cell signaling [19,23]. As shown in Figure 4, when the DCIS model (MCF10a-lined lumen with DCIS in the center) was patterned in the center channel of a tri-channel device and cocultured with HMFs in the side channels, invasion into the surrounding ECM was observed after 5 days. No invasion was observed when the normal mammary duct model (MCF10a-lined lumen) is cocultured with fibroblasts or when the DCIS lumen model is cultured alone. In control experiments, where MCF10a cells were added to the center of MCF10a-lined lumens in place of the DCIS cells, no invasion was observed in monoculture or in coculture with HMFs. Additionally, when DCIS cells were used to line the lumens in place of MCF10a cells, a confluent monolayer failed to form. We were able to count the number of invasive lesions per channel as well as per area, showing higher numbers of lesions in coculture to determine the invasiveness (Figures 4C and D).

**Figure 5:**
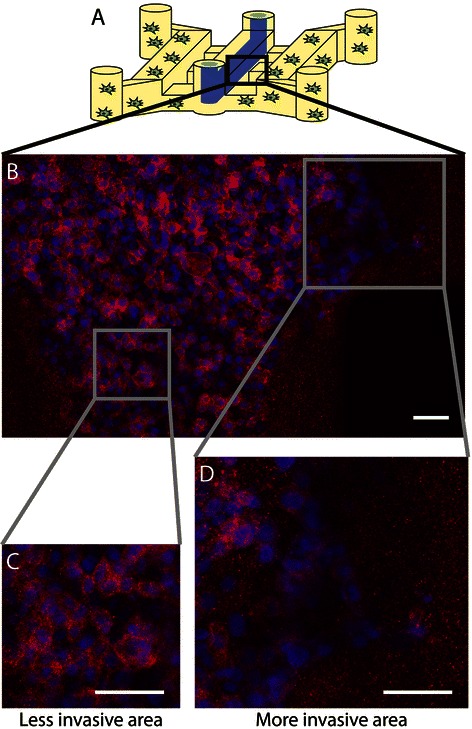
**Invasive lesions show decreased e-cadherin.** Schematic **(A)** and confocal slice **(B-D)** of bottom of DCIS model cocultured with HMFs for 5 days and stained for e-cadherin (red) and nuclei (blue). A magnified view of a less invasive region **(C)** compared to a more invasive region **(D)** indicate that e-cadherin is down regulated in cells that have undergone the invasive transition. Scale bars represent 50 μm.

**Figure 6:**
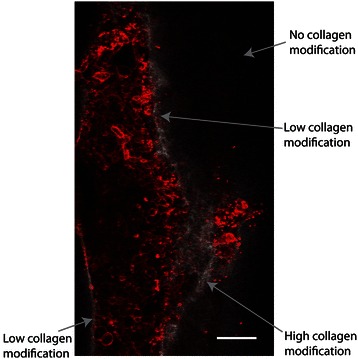
**Second Harmonic Generation (SHG) imaging of collagen modifications by invasive clusters.** Multiphoton image of a DCIS model cocultured with HMFs. Cells were stained with phalloidin to mark f-actin (shown in red). Using SHG imaging we are able to identify increased collagen I (show in white) modification near the invasive region compared to non-invasive regions. Scale bar represents 100 μm.

The invasive transition of DCIS in our lumen-based model was validated by multiple endpoint analyses. First, the invasive transition was analyzed by estimating the average number (Figure 4C) and area (Figure 4D) of invasive lesions sprouting out from the main lumen filled with DCIS cells, more similar to the measurement conducted for the transition in vivo. Second, the loss of adherens junctions in invasive MCF10aDCIS cells was examined. In cells undergoing transition from an epithelial to a more invasive cell type decreased e-cadherin levels are often observed [40]. When stained for e-cadherin, cells that have begun to invade into the surrounding matrix along the edge of the DCIS lumen model showed the decreased expression of e-cadherin compared to non-invading cells in the center (Figure 5). Third, we have also examined the changes in collagen architecture around MCF10aDCIS cells invading into stroma. Previous work has demonstrated that ECM alterations (i.e., increase of collagen density and changes in collagen fiber angles) are associated with increased breast cancer invasion [15,41]. Accordingly, we used multi-photon and second harmonic generation (SHG) imaging to investigate collagen I modifications around invasive DCIS lesions in our model. SHG is widely used to analyze the properties of ECM because the intensity of SHG is proportional to the density of collagen [15,42]. The compartmentalized fashion of our microsystem allows the imaging and analysis of the collagen structure associated with each cell type in coculture while minimizing inference from other cell types [15]. The SHG intensity in Figure 6 is approximately 2.5 times greater around the invasive DCIS lesion (indicated by the “high collagen modification” region) compared to the SHG intensity along the non-invasive region (indicated by the “low collagen modification” region) of the DCIS lumen model. This increased intensity indicates increased collagen I modification around the invasive lesion [15]. When performing SHG imaging, the power and gain were set so that the areas with “high collagen modification” would not be oversaturated, causing the areas with “no collagen modification” have a very low SHG intensity. SHG intensity was estimated using ImageJ. In future work, SHG imaging can be used to further investigate changes in collagen density and fiber alignment [41].

Here, we have developed and characterized an in vitro model of DCIS within a ductal structure and observed the effects of fibroblasts on invasion. The phenotype of invasive transition observed in the lumen system is analogous to the invasive phenotype observed in humans [43], in which cancer cells invade out from the mammary duct into the surrounding stroma. In contrast to common analysis methods of measuring the size and shape of in vitro DCIS cells cultured from a suspension of single cells, the quantification of lesion size from a duct-like structure is analogous to the quantification of tumor size and invasive margins used in humans [44]. Additionally, in vivo mouse studies of DCIS usually involve growing DCIS cells in the mammary fat pad [45,46], but cells are not limited to the ducts as in the MINO and MIND model. Together, these in vitro and in vivo methods have provided valuable insight into the signaling mechanisms involved in invasive transition. Our in vitro DCIS model with lumen-like structures will be a useful complementary model to currently available systems by providing a greater ability to isolate specific interactions than in vivo models and using techniques that are amenable for high-throughput screening when interfaced with automated pipetting systems [22,31,32]. This model enables myriad other studies looking at microenvironmental effects. In the present study we used collagen I and Matrigel, but it is possible to incorporate other ECM molecules, such as laminin-111 known for inducing epithelail call polarization [47], into the collagen itself or as a lumen coating [22,32,48]. Investigation of the relationship between DCIS and other stromal cell types, such as macrophages or adipocyte, is also possible in future work. Finally, the microchannel design used in this work has been previously used to study the effects of growth factor gradients on a microvessel model [23] and may enable the use of this model to investigate the influence of chemical gradients on DCIS invasion.

## Conclusions

In this study we have developed novel in vitro models of a normal mammary duct with physiologically relevant patterned lumen structures. By filling the lumens with DCIS cells we have developed a model to study the invasive transition of DCIS in response to microenvironmental factors. These models have 3D lumen geometries which are physiologically relevant and have been previously demonstrated to influence cell behavior. The viscous finger patterning method used in the generation of these models is user-friendly, requires only a micropipette to perform, and can be scaled up into large screening arrays using automated pipeting systems. Using this model of DCIS, we have demonstrated the ability to study the effects of HMFs and potentially other microenvironmental factors on the invasive transition of DCIS to IDC in a physiologically relevant in vitro setting. In future work, this platform can be expanded to study the role of other stromal cell types, ECM molecules, chemical gradients, and drug treatment. In addition, our microscale platform has significantly reduced the number of cells required for each endpoint, ultimately allowing multiple candidate biomarkers to be screened simultaneously with primary cells isolated from small biopsies from patients.

## Abbreviations

2D: Two-dimensional
3D: Three-dimensional
DCIS: Ductal carcinoma in situ
ECM: Extracellular matrix
HMF: Human mammary fibroblast
IDC: Invasive ductal carcinoma
PDMS: Polydimethylsiloxane
